# Methylthioadenosine Phosphorylase Genomic Loss in Advanced Gastrointestinal Cancers

**DOI:** 10.1093/oncolo/oyae011

**Published:** 2024-02-08

**Authors:** Natalie Y L Ngoi, Tin-Yun Tang, Catia F Gaspar, Dean C Pavlick, Gregory M Buchold, Emma L Scholefield, Vamsi Parimi, Richard S P Huang, Tyler Janovitz, Natalie Danziger, Mia A Levy, Shubham Pant, Anaemy Danner De Armas, David Kumpula, Jeffrey S Ross, Milind Javle, Jordi Rodon Ahnert

**Affiliations:** Department of Investigational Cancer Therapeutics, Division of Cancer Medicine, The University of Texas MD Anderson Cancer Center, Houston, TX, USA; Department of Haematology-Oncology, National University Cancer Institute, Singapore, Singapore; Division of Cancer Medicine, The University of Texas MD Anderson Cancer Center, Houston, TX, USA; Department of Investigational Cancer Therapeutics, Division of Cancer Medicine, The University of Texas MD Anderson Cancer Center, Houston, TX, USA; Foundation Medicine Inc., Cambridge, MA, USA; Department of Investigational Cancer Therapeutics, Division of Cancer Medicine, The University of Texas MD Anderson Cancer Center, Houston, TX, USA; Strathclyde Institute of Pharmacy and Biomedical Sciences, University of Strathclyde Glasgow, Glasgow, UK; Foundation Medicine Inc., Cambridge, MA, USA; Foundation Medicine Inc., Cambridge, MA, USA; Foundation Medicine Inc., Cambridge, MA, USA; Foundation Medicine Inc., Cambridge, MA, USA; Foundation Medicine Inc., Cambridge, MA, USA; Department of Gastrointestinal Medical Oncology, Division of Cancer Medicine, The University of Texas MD Anderson Cancer Center, Houston, TX, USA; Department of Gastrointestinal Medical Oncology, Division of Cancer Medicine, The University of Texas MD Anderson Cancer Center, Houston, TX, USA; Departments of Pathology, Urology and Medicine (Oncology), Upstate Medical University, Syracuse, NY, USA; Foundation Medicine Inc., Cambridge, MA, USA; Departments of Pathology, Urology and Medicine (Oncology), Upstate Medical University, Syracuse, NY, USA; Department of Gastrointestinal Medical Oncology, Division of Cancer Medicine, The University of Texas MD Anderson Cancer Center, Houston, TX, USA; Department of Investigational Cancer Therapeutics, Division of Cancer Medicine, The University of Texas MD Anderson Cancer Center, Houston, TX, USA

**Keywords:** MTAP loss, 9p21 loss, genomics, biomarkers, tumor, cholangiocarcinoma

## Abstract

**Background:**

One of the most common sporadic homozygous deletions in cancers is 9p21 loss, which includes the genes methylthioadenosine phosphorylase (*MTAP*), *CDKN2A*, and *CDKN2B*, and has been correlated with worsened outcomes and immunotherapy resistance. *MTAP*-loss is a developing drug target through synthetic lethality with MAT2A and PMRT5 inhibitors. The purpose of this study is to investigate the prevalence and genomic landscape of *MTAP-*loss in advanced gastrointestinal (GI) tumors and investigate its role as a prognostic biomarker.

**Materials and Methods:**

We performed next-generation sequencing and comparative genomic and clinical analysis on an extensive cohort of 64 860 tumors comprising 5 GI cancers. We compared the clinical outcomes of patients with GI cancer harboring *MTAP-*loss and *MTAP-*intact tumors in a retrospective study.

**Results:**

The prevalence of *MTAP*-loss in GI cancers is 8.30%. *MTAP*-loss was most prevalent in pancreatic ductal adenocarcinoma (PDAC) at 21.7% and least in colorectal carcinoma (CRC) at 1.1%. *MTAP*-loss tumors were more prevalent in East Asian patients with PDAC (4.4% vs 3.2%, *P* = .005) or intrahepatic cholangiocarcinoma (IHCC; 6.4% vs 4.3%, *P* = .036). Significant differences in the prevalence of potentially targetable genomic alterations (*ATM*, *BRAF*, *BRCA2*, *ERBB2*, *IDH1*, *PIK3CA*, and *PTEN)* were observed in *MTAP*-loss tumors and varied according to tumor type. *MTAP*-loss PDAC, IHCC, and CRC had a lower prevalence of microsatellite instability or elevated tumor mutational burden. Positive PD-L1 tumor cell expression was less frequent among *MTAP*-loss versus *MTAP*-intact IHCC tumors (23.2% vs 31.2%, *P* = .017).

**Conclusion:**

In GI cancers, *MTAP*-loss occurs as part of 9p21 loss and has an overall prevalence of 8%. *MTAP*-loss occurs in 22% of PDAC, 15% of IHCC, 8.7% of gastroesophageal adenocarcinoma, 2.4% of hepatocellular carcinoma, and 1.1% of CRC and is not mutually exclusive with other targetable mutations.

## Teaser

Methylthioadenosine phosphorylase (MTAP)-loss is a developing drug target through synthetic lethality with MAT2A and PMRT5 inhibitors. This study investigated the prevalence and genomic landscape of MTAP-loss in advanced gastrointestinal tumors and its role as a prognostic biomarker.

**Implications for Practice**

Methylthioadenosine phosphorylase (*MTAP*)-loss is an emerging biomarker for novel agents inhibiting MAT2A and PRMT5. This study found that 8% of gastrointestinal (GI) cancers have *MTAP*-loss. The high prevalence of *MTAP-*loss supports dedicated drug development of MAT2A and PRMT5 inhibitors in GI cancers. The lack of mutual exclusivity and the presence of actionable coalterations in *MTAP*-loss GI cancers indicate opportunities for combination or sequential therapeutic targeting in the future.

## Introduction

**Figure 1.**
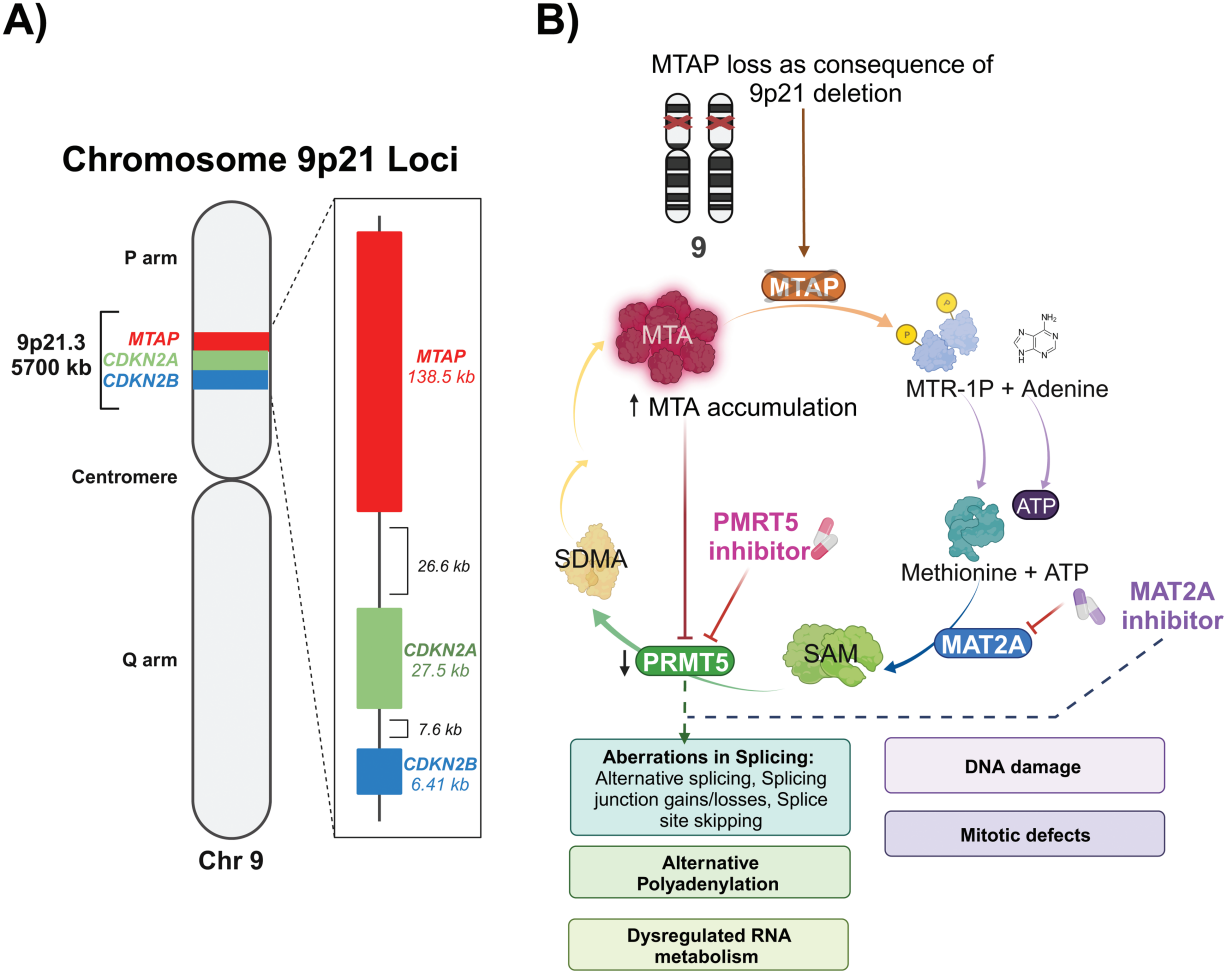
Genomic location of 9p21 and consequences of *MTAP*-loss.

Identifying genomic loci with recurring somatic homozygous deletions in cancer genomes has historically and recently been used as a strategy to identify new tumor suppressor genes.^1-3^ Cytogenetic studies in the 1990s demonstrated that the p21 region of chromosome 9 is a region with recurring homozygous deletions in multiple cancer types, which led to the discovery of the *CDKN2A* tumor suppressor gene in 1994.^4-6^ Homozygous deletion of tumor suppressor genes plays a key role in oncogenesis, and homozygous 9p21-loss (henceforth 9p21-loss) has been demonstrated to be an early evolutionary event in oncogenesis.^7,8^ The methylthioadenosine phosphorylase (*MTAP*) gene is immediately adjacent to *CDKN2A* within 9p21 and also frequently homozygously codeleted with *CDKN2A* and *CDKN2B* during a chromosomal interstitial deletion event—henceforth referred to as *MTAP*-loss.^9^ (Fig. 1A). From a pan-cancer study of data from The Cancer Genome Atlas, homozygous deletion of *MTAP* was described in 9.3% of cancers, with loss of heterozygosity at 9p21 due to hemizygous deletion of *MTAP* observed in another 27.8% of cancers.^10^ Despite the relatively high prevalence of 9p21/MTAP-loss, investigation in the field has been limited by the challenges associated with targeting loss-of-function mutations.

Interest and investigation over the biology of *MTAP-*loss has resurged following breakthrough work in 2016 that demonstrated *MTAP*-loss could be targeted through a synthetic lethal relationship with methionine adenosyltransferase 2A (MAT2A) and protein arginine methyltransferase 5 (PRMT5) inhibitors.^11-13^ MTAP is a rate-limiting enzyme controlling the final step of the methionine salvage pathway, which replenishes intracellular adenine and methionine pools, and plays a crucial role in rapidly proliferating and metabolically stressed tumor cells.^14^ (Fig. 1B) *MTAP*-loss causes an accumulation of methylthioadenosine (MTA), which has been associated with aggressive cancer phenotypes.^15,16^ Although the mechanism of action leading to synthetic lethality between *MTAP*-loss and MAT2A or PRMT5 inhibitors is yet to be definitively determined, early evidence suggests it could be due to increases in alternative splicing and polyadenylation.^17,18^ Importantly, proof-of-concept establishing that *MTAP*-loss can be targeted with PRMT5 inhibitors has been demonstrated in early phase clinical trials with a promising clinical efficacy signal.^19^

The potential role and impact of *MTAP*-loss as a therapeutic target in advanced gastrointestinal (GI) cancers have not yet been directly interrogated. Publications examining the genomic landscape and clinical impact of *MTAP*-loss are currently limited to the pan-cancer setting, with limited individual sample sizes in GI cancers. In a pan-cancer setting, *MTAP*-loss has been associated with shorter overall survival (OS).^10^ Importantly, 9p21/*MTAP*-loss is also an emerging predictive biomarker of an immunogenically “cold” tumor microenvironment and associated with distinct shifts in intra-tumoral immune cell abundance, reduced T-cell receptor repertoire diversity, reduced PD-L1 positivity, and changes in immunomodulatory gene expression.^10,20^ Across clinical cohorts treated with anti-PD(L)1 immunotherapy and chiefly comprised of patients with melanoma, lung, and urothelial cancer, significantly reduced progression-free (PFS) and disease-specific survival were observed for patients with tumors harboring 9p21-loss versus 9p21-wildtype.^10^ In advanced GI cancers though, both the prevalence, genomic landscape, and clinical characteristics of *MTAP*-loss have not been previously examined. The purpose of this study is to describe the prevalence of *MTAP-*loss in GI cancers and examine differences in coalterations between *MTAP*-loss and *MTAP*-intact cancers. We also examined potential differences in immune biomarkers and the prognostic clinical implications of *MTAP*-loss in common GI cancer types.

## Methods

### Patient Cohorts and Clinical Characteristics

#### Genomics-Only Cohort (*N* = 64 860)

Approval for this study was obtained from the Western Institutional Review Board (Protocol No. 20152817). For this cross-sectional study, betweenJanuary 1, 2018 and July 15, 2022, patients with any of 5 histologically defined GI cancer types (pancreatic ductal adenocarcinoma [PDAC], intrahepatic cholangiocarcinoma [IHCC], hepatocellular carcinoma [HCC], colorectal carcinoma [CRC], and gastroesophageal adenocarcinoma [GEAC]), who had previously undergone comprehensive genomic profiling at a Clinical Laboratory Improvement Amendments (CLIA)-certified and College of American Pathologists (CAP)-accredited reference molecular laboratory (Foundation Medicine, Inc.) as part of their clinical care, were selected. Tumor profiles were identified based on the histological subtype submitted by physicians and validated by central pathology review. Clinicopathological data including patient age and gender, routine histology and immunohistochemical staining results, and confirmation of the diagnosis were extracted from medical records and pathology reports.

#### Clinical Outcomes Cohort (*N* = 102)

To determine the clinical significance of homozygous *MTAP*-loss among patients with GI cancer treated at The University of Texas MD Anderson Cancer Center (MDACC), we identified 102 patients diagnosed with advanced PDAC and IHCC between January 11, 2018 and August 2, 2022 and whose tumors had defined *MTAP* status (-loss or -intact), determined by copy number status via next-generation sequencing (NGS). Patients in *MTAP-*intact and homozygous-loss groups were matched by age, gender, and ethnicity. Clinical information, including demographic information, treatment history, and response to treatment, were retrieved from a retrospective medical record review. PFS was defined as the time of initiation of any line of systemic therapy to the date of clinical or radiological disease progression/treatment discontinuation on that line of therapy, as determined by the treating physician. OS was defined as the time of diagnosis to death from any cause.

### Comprehensive Genomic Profiling

Comprehensive genomic profiling for the Genomics-only cohort was performed on United States Food and Drug Administration (U.S. FDA)-approved hybridization-captured, adaptor ligation–based libraries using DNA extracted from formalin-fixed paraffin-embedded tumor. All samples forwarded for DNA extraction contained a minimum of 20% tumor cells. The samples were assayed using adaptor-ligation and hybrid capture NGS for all coding exons from up to 324 cancer-related genes, plus select introns from up to 31 genes frequently rearranged in cancer. Patient samples were sequenced and evaluated for genomic alterations, including base substitutions, insertions, deletions, copy number alterations (amplifications and homozygous deletions), and for select gene fusions/rearrangements, as previously described.^21^ The bioinformatics processes used in this study included Bayesian algorithms to detect base substitutions, local assembly algorithms to detect short insertions and deletions, a comparison with process-matched normal control samples to detect gene copy number alterations, and an analysis of chimeric read pairs to identify gene fusions as previously described.^22^ Unless otherwise specified, short variants (single nucleotide variants and short insertion/deletions) were included in the analysis if they were annotated as either “Known Pathogenic” or “Likely Pathogenic.” *MTAP*-intact or loss status was determined using NGS, utilizing a copy number assessment algorithm, which is a component of this assay. All cases reported as *MTAP*-loss featured an *MTAP* copy number of zero indicating homozygous loss.

### PD-L1 Immunohistochemistry

PD-L1 immunohistochemistry (IHC) was performed on subsets of tumors in the Foundation Medicine cohort using the Dako 22C3 PharmDx assay in a CLIA- and CAP-accredited reference laboratory, per manufacturer’s instructions. Interpretation of PD-L1 IHC was performed by a board-certified pathologist to determine the tumor proportion score (TPS), which is defined as the number of PD-L1 staining tumor cells with any convincing partial or complete linear membrane staining of viable tumor cells distinct from cytoplasmic staining, divided by the total number of viable tumor cells, multiplied by 100. PD-L1 “low positive” was defined as TPS scores of 1%-49%, while PD-L1 “high positive” was defined as TPS scores > 50%.

### Tumor Mutational Burden

Tumor Mutational Burden (TMB) in the Genomics-only cohort was determined on 0.83-1.14 megabases (Mb) of sequenced DNA using a mutation burden estimation algorithm that, based on the genomic alterations detected, extrapolates to the exome or the genome as a whole as previously described.^23^ Assessment of microsatellite instability (MSI) was performed from DNA sequencing across 114 loci, as previously described. Each microsatellite locus had a repeat length of 7-39 base pairs. The NGS-based “MSI score” was translated into categorical MSI-high, MSI-intermediate, or microsatellite stable by unsupervised clustering of specimens for which MSI status was previously assessed via gold standard methods.^24^

### Genomic Ancestry

Our data in the Genomics-only cohort lacked patient- or physician-reported race, thus patient ancestry was inferred using ancestry-informative markers to classify genomic ancestry. A random forest classifier was used to identify genomic ancestry using genetic variation at single nucleotide polymorphism sites to assign patient samples to one of the ancestral groups—East Asian, European, South Asian, African, Admixed American, and South Asian.^25^

### COSMIC Trinucleotide Mutational Signatures

Determination of mutational signatures was performed as previously described.^26^ The distribution of mutational burden was used to identify a suitable threshold for the identification of mutational signatures.^27^ We focused on 6 main signatures in this work: mismatch repair (signatures 6, 15, 20, 26), apolipoprotein B mRNA editing catalytic polypeptide-like (APOBEC; signature 2, 13), ultraviolet radiation (UV; signature 7), polymerase epsilon (signature 10), tobacco smoking (signature 4), and alkylating agents (signature 11).

### Statistical Analyses

Statistical analyses were performed using SPSS 28.1.1 (IBM Corp., Armonk, NY, USA). Categorical variables were compared using Fisher’s exact test to determine proportional differences between groups. False discovery rate was corrected using Bonferonni’s correction. All statistical tests were 2-sided, and a *P*-value <.05 was considered significant. Data visualization was performed using R 4.3.1. For survival analysis including OS and PFS, the log-rank test was used to calculate *P*-values between patient groups, and the Kaplan-Meier method was used to plot survival curves with Prism 10. Cox proportional hazards regression (HR) model was used to conduct multivariate analysis of survival and to calculate the hazard ratio, 95% CI, and associated *P*-values.

## Results

### Genomics-Only Cohort

**Figure 2.**
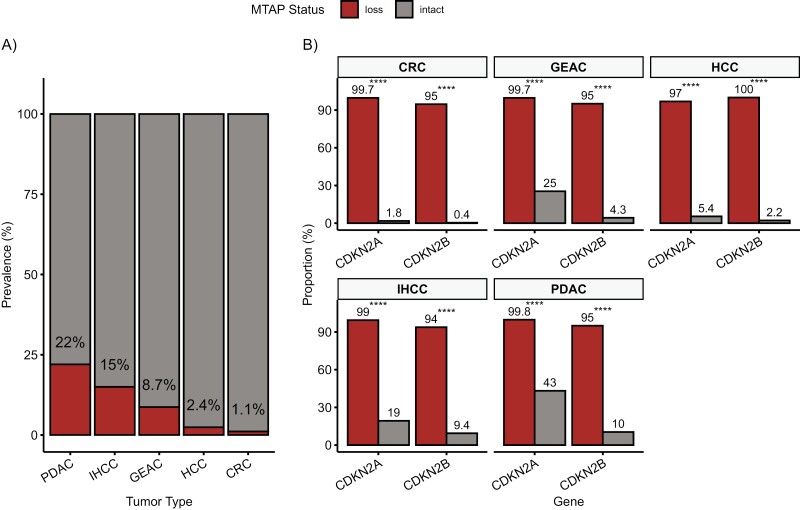
Prevalence of *MTAP*-loss and 9p21 in GI cancers. (**A**) The prevalence of *MTAP*-loss by IHC in different GI cancers. (**B**) Coalteration rates of 9p21-loss related genes, *CDKN2A and CDKN2B*, in *MTAP-*loss and *MTAP*-intact. Abbreviations: PDAC, pancreatic ductal adenocarcinoma; IHCC, intrahepatic cholangiocarcinoma; CRC, colorectal carcinoma; GEAC, gastroesophageal adenocarcinoma; HCC, hepatocellular carcinoma. *P ≤ .05; **P ≤ .01; ***P ≤ .001; ****P ≤ .0001.

*MTAP*-loss occurred in 21.6% (3401/12 319 tumors) of PDAC, 15.3% (785/4352 tumors) of IHCC, 8.7% (589/6143 tumors) of GEAC, 2.4% (32/1306 tumors) of HCC, and 1.1% (396/35 537 tumors) of CRC (Fig. 2A; Supplementary Fig. S1). *MTAP*-loss overall occurred in 8.7% of the 5 GI tumors profiled (5203/59 657 tumors) and was balanced in patient age and gender (Table 1). Most tumors were of European genetic ancestry across all tumor types (PDAC: 78.2%; IHCC: 73.7%; GEAC: 90.7%; HCC: 65.3%; CRC: 72.4%). However, more *MTAP*-loss tumors were observed to be of East Asian (EAS) genomic ancestry within PDAC (4.4% vs 3.2%, *P* = .005) and IHCC (6.4% vs 4.3%, *P* = .036) tumor groups, compared with *MTAP*-intact.

**Table 1.** Baseline patient characteristics in Genomics-only and Clinical outcomes cohorts.

Cohort	Tumor type and *MTAP* status									
	PDAC		IHCC		HCC		CRC		GEAC	
	Intact	Loss	Intact	Loss	Intact	Loss	Intact	Loss	Intact	Loss
Genomics-only cohort^a^										
Number of case (% within tumor type)	12 319 (78.4)	3401 (21.6)	4352 (84.7)	785 (15.3)	1306 (97.6)	32 (2.4)	35 537 (98.9)	396 (1.1)	6143 (91.3)	589 (8.7)
Gender (% male)	52.9	52.4	48.9	51.1	75.3	59.4	55.6	58.3	86.4	83.7
Age, median, years (range)	67 (23-89)	67 (25-89)	66 (18-89)	66 (18-89+)	67 (5-89)	61.5 (34-83)	61 (10-89)	61.5 (21-89)	66 (22-89)	65 (21-89)
Genomic ancestry, %										
AFR	10.1	9.8	8.8	8.0	13.9	12.5	12.5	10.9	2.8	2.0
AMR	7.5	80.9	11.9	10.8	12.5	12.5	10.1	10.9	5.1	5.6
EAS	3.2	4.4	4.3	6.4	6.7	9.4	40.5	45.5	1.1	0.8
EUR	78.5	77.1	73.6	74.2	65.3	65.6	72.4	73.5	90.6	91.0
SAS	0.7	0.7	1.4	0.5	1.6	0	0.9	0.3	0.4	0.5
Clinical outcomes cohort										
Number of cases (% within tumor type)	11	21	49	21						
Gender (% male)	72.7	33.3	50.0	33.3						
Age, median, years (range)	62.7 (52.4-74.4)	60.8 (19.0-70.9)	57.3 (23.6-77.2)	54.0 (23.3-80.5)						
Ethnicity										
Caucasian	9	14	40	17						
African	1	5	1	1						
Hispanic	1	2	3	1						
Asian	0	0	5	2						
NGS panel used, %										
FoundationOne CDx	5	17	43	21						
Perthera	2	1	0	0						
Tempus xT	3	3	6	0						
MSK IMPACT	1	0	0	0						

^a^The FoundationOne CDx NGS panel was used for the entire Genomics-only cohort: 64 860 (100%).

Abbreviations: *MTAP*, methylthioadenosine phosphorylase; PDAC, pancreatic ductal adenocarcinoma; IHCC, intrahepatic cholangiocarcinoma; HCC, hepatocellular carcinoma; CRC, colorectal carcinoma; GEAC, gastroesophageal adenocarcinoma; AFR, African; AMR, admixed American; EAS, East Asian; EUR, European; SAS, South Asian.

Comparative genomic analysis between *MTAP*-loss and *MTAP*-intact mutation profiles in each GI cancer demonstrated near universal concomitant alterations in both *CDKN2A* and *CDKN2B* in *MTAP*-loss tumors (Fig. 2B). All of the 5 GI cancers had statistically different prevalence of *CDKN2A* and *CDKN2B* coalterations according to *MTAP* status. For example, of the 35 537 CRC tumors that were *MTAP*-intact, only 1.8% and 0.4% of them demonstrated mutations in *CDKN2A* and *CDKN2B*, respectively. The overall prevalence of *MTAP*-loss in CRC is low at 1.1%, but notably 99.7% and 95% of these cases have coalterations in *CDKN2A* and *CDKN2B*. In comparison, *MTAP*-intact PDAC cases have a high prevalence of *CDKN2A* and *CDKN2B* mutations at 43% and 10%, respectively. *MTAP-*loss PDAC cases occur near universally as part of 9p21 loss, with concomitant *CDKN2A* and *CDKN2B* coalterations in 99.8% and 95% of cases, respectively.

**Figure 3.**
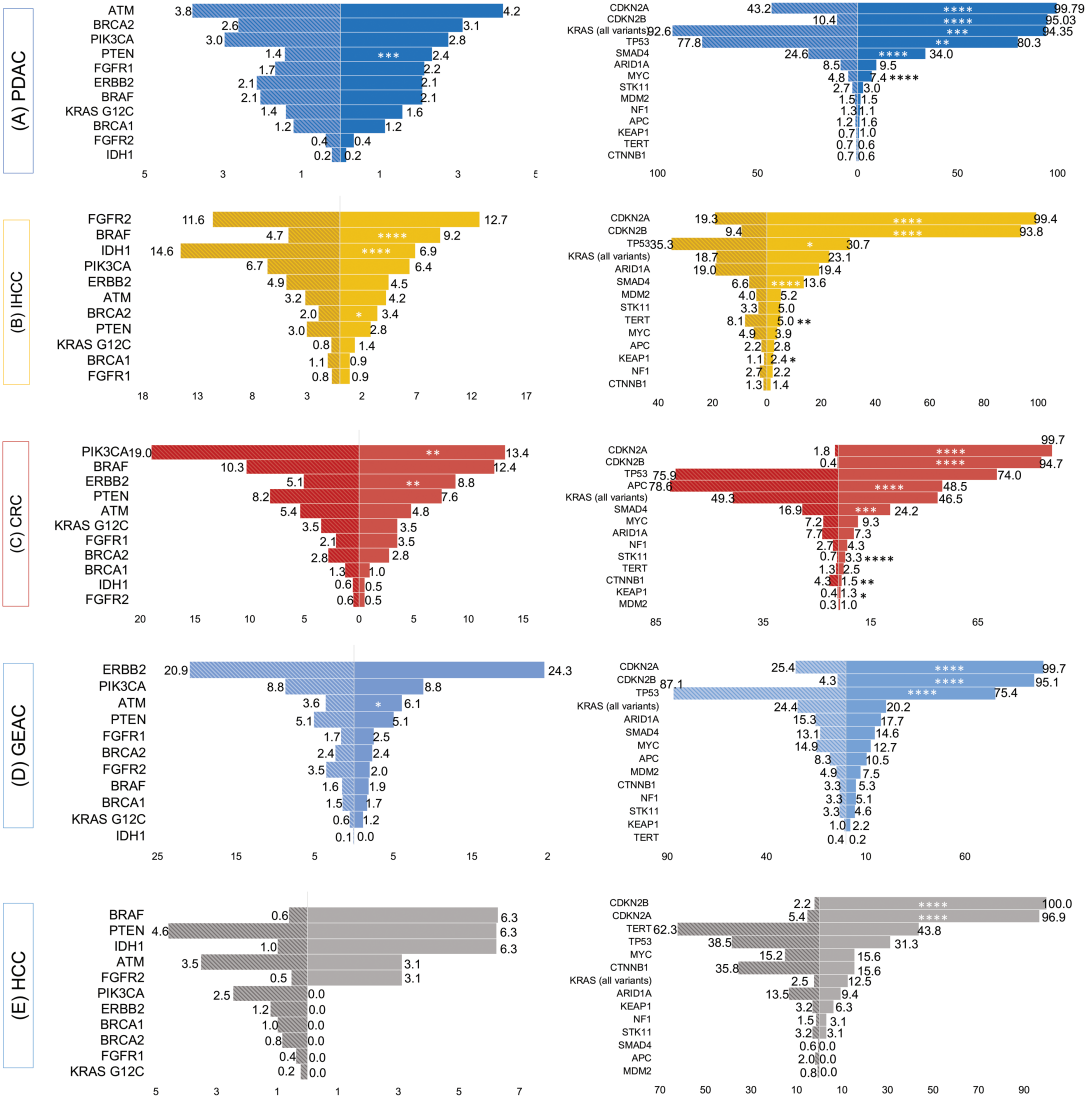
Comparison of differences in genomic alterations between *MTAP*-loss and *MTAP*-intact tumors in the Genomics-only cohort. The distribution of co-occurring potentially targetable and un-targetable genomic alterations in advanced (**A**) pancreatic ductal adenocarcinoma (PDAC), (**B**) intrahepatic cholangiocarcinoma (IHCC) (**C**) colorectal carcinoma (CRC), (**D**) gastroesophageal adenocarcinoma (GEAC), and (**E**) hepatocellular carcinoma (HCC) are shown. Striped bars represent MTAP-intact groups, solid bars represent MTAP-loss groups, and numbers represent (%) within tumor group. *P ≤ .05; **P ≤ .01; ***P ≤ .001; ****P ≤ .0001.

Among genomic alterations deemed potentially targetable, whereby an approved targeted therapy exists in the same or other tumor types, statistically significant differences were observed between *MTAP*-loss versus *MTAP*-intact profiles (Fig. 3, Supplementary Fig. S2). In CRC, *ERBB2* (8.8% vs 5.1%, *P* = .008) alterations were more frequently observed while *PIK3CA* (13.4% vs 19.0%, *P* = .015) alterations were less frequently observed in homozygous *MTAP*-loss compared with MTAP-intact tumors. In GEAC, *ATM* (6.1% vs 3.6%, *P* = .038) alterations were more frequent among homozygous *MTAP*-loss tumors. No statistically significant differences were detected in HCC. In IHCC, homozygous *MTAP*-loss tumors had a higher frequency of pathogenic mutations in *BRCA2* (3.4% vs 2.0%, *P* = .043) and *BRAF* (9.2% vs 4.7%, *P* < .0001) but had a lower frequency of pathogenic mutations in *IDH1* (6.9% vs 15%, *P* < .0001), compared with *MTAP*-intact tumors. Pertinent negatives for IHCC also include no difference in the frequency of *FGFR2* alterations (12.7% vs 11.6%, *P* = .594). Finally, in PDAC deleterious mutations in *PTEN* were more frequently found in homozygous *MTAP*-loss tumors (2.4% vs 1.4%, *P* = .001).

Differences in the frequencies of observed alterations among other ‘undruggable’ cancer genes varied according to tumor type (Fig. 3, Supplementary Fig. S2). *MTAP*-loss CRC was associated with a lower frequency of alterations in *APC* (48% vs 79%, *P* < .0001) and *CTNNB1* (1.5% vs 4.3%, *P* = .014), but a higher frequency of alterations in *STK11* (3.3% vs 0.7%, *P* < .0001) and *KEAP1* (1.3% vs 0.4%, *P* = .067) compared with *MTAP*-intact CRC. In GEAC, *MTAP*-loss was associated with a lower frequency of alterations in *TP53* (75% vs 87%, *P* < .0001*). MTAP*-loss IHCC was associated with slightly lower coalterations in *TP53* (31% vs 35%, *P* < .05) and *TERT* (5% vs 8.1%) and higher coalterations in *KRAS* (23% vs 19%, *P* < .05), *SMAD4* (14% vs 6.6%, *P* < .0001), and *KEAP1* (2.4% vs 1.1%, *P* < .05). *MTAP-*loss PDAC was associated with a higher frequency of alterations in *KRAS* (94.4% vs 92.6%, *P* = .001), *TP53* (80.3% vs 77.8%, *P* = .005), and *MYC* (7.4% vs 4.8%, *P* < .0001), compared with *MTAP*-intact PDAC. Across CRC, IHCC, and PDAC, *SMAD4* alterations were statistically more prevalent among homozygous *MTAP*-loss versus *MTAP*-intact GI cancers (PDAC [34.1% vs 24.6%, *P* < .0001]; IHCC [13.6% vs 6.6%, *P* < .0001]; CRC [24.2% vs 16.9%, *P* = .001]).

**Figure 4.**
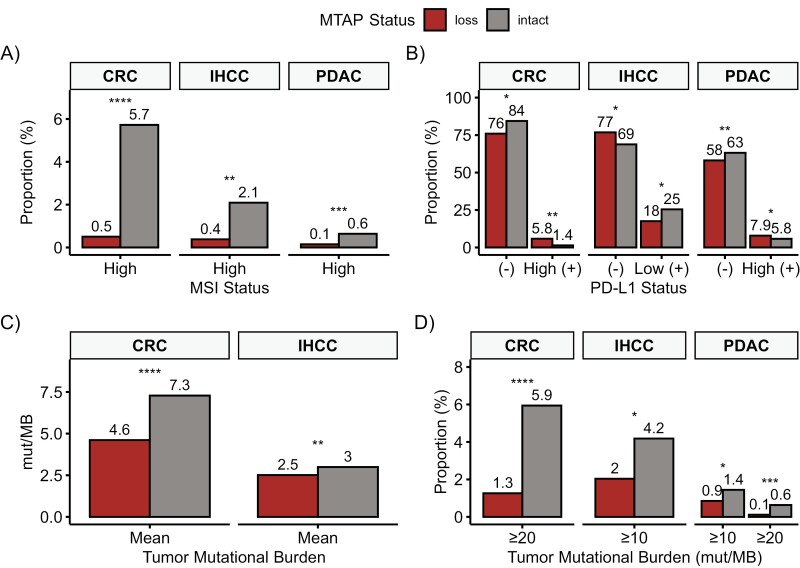
Comparative analysis of immunotherapy markers between *MTAP*-loss and *MTAP*-intact tumors. Only statistically significant findings are shown and includes colorectal carcinoma (CRC), intrahepatic cholangiocarcinoma (IHCC), and pancreatic ductal adenocarcinoma (PDAC). (**A**) MSI analysis, (**B**) PD-L1 analysis, (**C**) mean tumor mutation burden, and (**D**) tumor mutation burden at commonly used cutoffs. *P ≤ .05; **P ≤ .01; ***P ≤ .001; ****P ≤ .0001. (-) is Negative, (+) is Positive.

In predictive immunotherapy biomarkers, statistically significant differences by MTAP status were seen only in CRC, IHCC, and PDAC. *MTAP*-loss tumors had a lower prevalence of concomitant MSI-H in CRC (0.5% vs 5.7%, *P* < .0001), IHCC (0.4% vs 2.1%, *P* = .001), and PDAC (0.1% vs 0.6%, *P* = .0008; Fig. 4A). Thirty-four percentage of PDAC, 17.5% of IHCC, 25% of HCC, 18.2% of CRC, and 12.3% of GEAC tumors had available PD-L1 status by IHC and differences varied by tumor type (Fig. 4B; Supplementary Table S1). The mean TMB was significantly lower in *MTAP*-loss in CRC (4.6 vs 7.3 Mut/Mb, *P* < .0001) and IHCC (2.5 vs 3.0 Mut/Mb, *P* = .0015; Fig. 4C). Significant findings according to TMB thresholds were also seen in CRC, IHCC, and PDAC (Fig. 4D).

COSMIC trinucleotide genomic signatures were analyzable from 5234 genomic profiles within the Genomics-only cohort (Supplementary Table S2). APOBEC enzyme trinucleotide signature was more frequently observed among *MTAP*-loss CRC (7.3% vs 1.3%, *P* = .054) and PDAC (16.7% vs 5.6%, *P* = .044), compared with *MTAP*-intact tumors; while UV radiation signature was more frequently observed among homozygous *MTAP*-loss CRC compared with *MTAP*-intact tumors (4.9% vs 0.2%, *P* = .015).

A summary table of statistically significant coalterations and immunotherapy markers in *MTAP*-loss GI cancers is included as Table 2.

**Table 2.** Summary of genomic coalterations and immunotherapy markers in MTAP-loss GI cancers.

Tumor	Higher prevalence in *MTAP*-loss		Lower prevalence in *MTAP*-loss	
	Genomic alterations	Immunotherapy markers	Genomic alterations	Immunotherapy markers
CRC	*CDKN2A*, *CDKN2B*, *ERBB2*, *KEAP1*, *SMAD4*, *STK11*	PD-L1 high positive	*APC*, *CTNNB1*, *PIK3CA*	MSI-high, mean TMB, TMB ≥ 20 mut/Mb, PD-L1 negative
GEAC	*ATM*, *CDKN2A*, *CDKN2B*, *KRAS (G12X)*	—	*TP53*	—
HCC	*CDKN2A*, *CDKN2B*	—	—	—
IHCC	*BRAF*, *BRCA2*, *CDKN2A*, *CDKN2B*, *KEAP1*, *KRAS* (all variants), *SMAD4*	PD-L1 negative	*IDH1*, *TERT*, *TP53*	MSI-high, mean TMB, TMB ≥ 10 mut/Mb, PD-L1 low positive
PDAC	*CDKN2A*, *CDKN2B*, *KRAS* (all variants), *MYC*, *PTEN*, *SMAD4*, *TP53*	PD-L1 high positive	—	MSI-high, TMB ≥ 10 mut/Mb, TMB ≥ 20 mut/Mb, PD-L1 negative

### Clinical Outcomes Cohort

**Figure 5.**
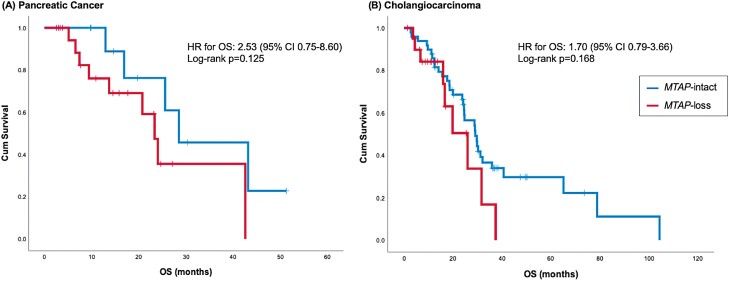
Effect of *MTAP* status on overall survival in the Clinical outcomes cohort. (**A**) Kaplan-Meier curve for overall survival in patients with advanced pancreatic cancer. (**B**) Kaplan-Meier curve for overall survival in patients with advanced cholangiocarcinoma.

Forty-two patients with advanced stage PDAC (*n* = 21) or IHCC (*n* = 21) with *MTAP*-loss were identified from MDACC and were compared against 60 matched patients who were *MTAP*-intact (Table 1; Supplementary Table S3). In PDAC or IHCC, *MTAP*-loss was associated with a numerically shorter overall survival (OS) compared with *MTAP*-intact. However, these findings were not statistically significant (Fig. 5). On multivariate analysis by coalterations, *CDKN2A* (HR 2.15, 95% CI 1.06-4.40, *P* = .035), *CCNE1* (HR 8.86, 95% CI 1.16-67369, *P* = .035), and *MYC* (HR 1.28, 95% CI 1.28-7.10, *P* = .012) were associated with worse OS in patients with advanced IHCC (Supplementary Table S4).

## Discussion

*MTAP*-loss is a novel and emerging therapeutic target. Accumulation of MTA in *MTAP*-loss cancer cells sensitizes them to additional PMRT5 and MAT2A inhibition.^12,28^ This synthetic lethal relationship is now actively being exploited as a method of targeting *MTAP*-loss in early phase clinical trials enrolling advanced solid tumors. A Phase I trial of MRTX1719, a PRMT5 inhibitor, has led to RECIST partial responses in *MTAP*-loss mesothelioma, non-small cell lung cancer, melanoma, gallbladder adenocarcinoma, and malignant peripheral nerve sheath tumor.^19^ Notably, the early efficacy signal in gallbladder adenocarcinoma, which is frequently an aggressive cancer and resistant to therapies, raises great excitement and interest in investigating *MTAP*-loss for GI cancers.

As an evolving therapeutic target though, little is known regarding *MTAP*-loss in GI cancers—including basic epidemiological data such as prevalence and genomic coalterations, which can influence clinical trial feasibility decisions. Here, we undertook a cross-sectional study of >64 000 tumor profiles across the 5 most common GI cancer types to establish the prevalence of *MTAP*-loss, describe genomic differences between *MTAP*-loss and *MTAP*-intact tumors, and delineate any intersection with established immunotherapy markers. To our knowledge, this represents the largest study investigating genomic profiles by *MTAP* status in GI cancers.

We demonstrated that *MTAP-*loss is potentially one of the most prevalent targetable mutations in PDAC, IHCC, and GEAC, with prevalence of 22%, 15%, and 8.7%, respectively.^29-31^*MTAP*-loss is extremely uncommon in HCC and CRC though, making it unlikely that clinical trials of *MTAP-*loss will specifically seek to enroll patients with these cancers. Regardless of absolute prevalence though, an important finding in our study is that in GI cancers *MTAP-*loss occurs almost exclusively as part of 9p21 loss with codeletions of *CDKN2A* and *CDKN2B*. Prior genomic studies and even commercial panels have not always tested specifically for *MTAP*-loss, making it challenging to perform retrospective studies on clinical outcomes of patients with tumors harboring *MTAP*-loss.^32,33^ Testing for *CDKN2A* and *CDKN2B* is near ubiquitous though and this finding allows us to extrapolate the presence of *MTAP*-loss or perform dedicated testing for *MTAP*-loss in select scenarios. There is also growing literature on the molecular and clinical impact of 9p21-loss, and the recognition that *MTAP*-loss is synonymous to 9p21-loss in GI cancers facilitates a better understanding of evolving literature.^10,20,34^ In GEAC and PDAC, however, there is a proportion of *MTAP*-intact tumors with *CDKN2A* and *CDKN2B* alterations, making confirmation of *MTAP*-loss in these tumor types important.

No significant differences were observed in the demographic features (age or gender) of *MTAP*-loss patients with GI cancer. However, our study found that patients with East Asian genomic ancestry were more likely to have *MTAP*-loss. This could potentially imply an underlying genetic risk with implications on clinical need, such as that seen in EGFR mutations and lung cancers in East Asian patients.^35^ Additional study is needed though, as there are conflicting observations in the existing literature with other studies supporting both increases and decreases in the prevalence of *MTAP-*loss in East Asian patients.^36,37^

Our study does not find mutual exclusivity with other targetable driver mutations in GI cancers with *MTAP*-loss. Although current *MTAP*-loss clinical trials investigate MAT2A or PRMT5 inhibitors as monotherapy, combination targeted therapy may ultimately prove more efficacious.^38^ The detailed mutational profiling in our study establishes the prevalence of coalterations that may be amenable to combination therapy. For example, in IHCC, we found that *MTAP*-loss was associated with a significantly increased prevalence of *BRCA2* and *BRAF* coalterations. This potentially suggests a biological codependency that could be exploited with combination therapeutics. A previous study associated breast cancer risk with defects in methionine metabolism and a methionine-dependence phenotype in *BRCA1/2* mutation carriers.^39^ These intriguing links between *BRCA2* mutation status with metabolic dependency warrant further preclinical study to establish a potential rational combination therapy of poly(ADP)-ribose polymerase inhibitors and MAT2A or PRMT5 inhibitors in *MTAP*-loss GI cancers. In PDAC, mutations in *PTEN* were enriched in *MTAP*-loss and numerous early phase clinical trials investigating novel PI3K/Akt/mTOR-targeted therapies may provide future candidates for combination therapy.^40^ Alternatively, a sequential targeting approach could be envisioned for patients with tumors harboring *MTAP*-loss as well as targetable genomic coalterations, thus increasing the number of lines of targeted therapy available to such patients. For example, 36% of *BRAF*-mutated IHCC has previously been described to be coaltered with *MTAP*-loss, which our current study re-capitulates with an enrichment of *BRAF* mutations in *MTAP*-loss IHCC.^41^ These patients with both *BRAF* mutations and *MTAP*-loss would be candidates to sequence *BRAF* inhibitors, which have tumor-agnostic FDA approval, with a clinical trial of PRMT5 or MAT2A inhibitors on progression, and vice versa.^42^

9p21 loss and/or *MTAP*-loss has previously been associated with poor response to cancer immunotherapy and hypothesized to be related to immune evasion via cell cycle, metabolic, and type I interferon response pathways.^43^ In the Genomics-only cohort, we found that *MTAP*-loss CRC, IHCC, and PDAC were significantly less likely to harbor MSI-H. PD-L1 expression was also significantly lower in *MTAP*-loss IHCC but surprisingly higher in PDAC and CRC. However, *MTAP*-loss CRC was also associated with a higher frequency of alterations in *STK11* and *KEAP1*, which have been linked to immunotherapy resistance in NSCLC and pan-cancer cohorts.^44,45^

Mutational signatures are thought to infer a tumor’s mutational fingerprints and elaborate on multiple cancer processes involved in tumor initiation and progression.^27,46^ In the Genomics-only cohort, we found increased APOBEC and UV mutational signatures among *MTAP-*loss PDAC and CRC, respectively. This finding may suggest differences in endogenous and exogenous mutational signatures in *MTAP*-loss GI cancers and further investigation is warranted.

Finally, no clinical data exist regarding the survival outcomes of patients with GI cancer harboring *MTAP*-loss. In both advanced PDAC and IHCC, patients with tumors harboring *MTAP*-loss had a shorter median OS compared with those whose tumors were *MTAP*-intact, although these findings did not meet statistical significance. In IHCC, the presence of *CDKN2A* alteration, but not *MTAP*-loss was associated with significantly worse median OS on multivariate analysis. Further prospective data are required to confirm the prognostic clinical significance of *MTAP*-loss in advanced PDAC and IHCC.

Limitations of our study include a small patient cohort size with clinical outcomes (*n* = 102) as well as approximately only one-third and <10% of the Genomics-only cohort having PD-L1 IHC and COSMIC trinucleotide mutational signature analysis performed. Additional limitations include those inherent to retrospective studies including risk of selection bias as well as significant heterogeneity in treatment, surveillance, and follow-up among patients included in the Clinical outcomes cohort. Although our study used standardized methods (NGS) for the detection of homozygous *MTAP*-loss in both cohorts, epigenetic silencing of *MTAP* via aberrant promoter methylation is another recognized mechanism for *MTAP* inactivation in melanoma and glioblastoma, which was not explored in the present study.^47,48^

## Conclusions

In conclusion, *MTAP*-loss is an emerging therapeutic target with a high prevalence in PDAC, IHCC, and GEAC and unique mutational profiles. *MTAP*-loss occurs as part of 9p21-loss in GI cancers and our genomic and immunotherapy marker profiling lays the groundwork for future studies in the field. The lack of mutual exclusivity and identification of actionable coalterations in *MTAP*-loss GI cancers suggests opportunities for sequential or combination therapeutic approaches.

## Supplementary Material

oyae011_suppl_Supplementary_Figures_1-2

oyae011_suppl_Supplementary_Tables_1-4

## Data Availability

The datasets generated and/or analyzed during the study are available from the corresponding author, on request.

## Previous Presentations

This study was awarded the Conquer Cancer Merit Award by Conquer Cancer The ASCO Foundation at the ASCO Gastrointestinal Cancers Symposium 2022, San Francisco, and at the 2022 ASCO Annual Meeting, Chicago.
