# Supplementary material for: Methylthioadenosine Phosphorylase Genomic Loss in Advanced Gastrointestinal Cancers
Source: Oncologist. 2024 Feb 8;29(6):493–503. doi: 10.1093/oncolo/oyae011 (PMC11144995; doi:10.1093/oncolo/oyae011)
Supplement: oyae011_suppl_Supplementary_Figures_1-2 [file oyae011_suppl_supplementary_figures_1-2.docx]

**Supplementary Figures:**

**Supplementary Figure 1: Distribution of Gastrointestinal Cancer Subtypes by *MTAP*-loss/intact status within Genomics-only cohort.** *Legend: Pancreatic ductal adenocarcinoma (PDAC); Intrahepatic cholangiocarcinoma (IHCC); Hepatocellular carcinoma (HCC); Colorectal carcinoma (CRC); Gastroesophageal adenocarcinoma (GEAC).*

**Supplementary Figure 2: Comparison of statistically significant differences in genomic alterations between *MTAP*-loss and -intact tumors in the Genomics-only cohort.** Only statistically significant findings are shown and includes Colorectal carcinoma (CRC), Gastroesophageal adenocarcinoma (GEAC), Hepatocellular carcinoma (HCC), Intrahepatic cholangiocarcinoma (IHCC) and Pancreatic ductal adenocarcinoma (PDAC). Statistically significant findings in genes other than *CDKN2A* and *CDKN2B*. *Legend: Red represents MTAP-loss and Gray MTAP-intact. * P ≤ 0.05; ** P ≤ 0.01; *** P ≤ 0.001; **** P ≤ 0.0001*
