# Supplementary material for: Methylthioadenosine Phosphorylase Genomic Loss in Advanced Gastrointestinal Cancers
Source: Oncologist. 2024 Feb 8;29(6):493–503. doi: 10.1093/oncolo/oyae011 (PMC11144995; doi:10.1093/oncolo/oyae011)
Supplement: oyae011_suppl_Supplementary_Tables_1-4 [file oyae011_suppl_supplementary_tables_1-4.docx]

**Supplementary Tables**

**Supplementary Table 1: PD-L1 positivity according to MTAP tumor status in Genomics only cohort**

|  | **PDAC** | | | **IHCC** | | | **HCC** | | | **CRC** | | | | **GEAC** | | |
| --- | --- | --- | --- | --- | --- | --- | --- | --- | --- | --- | --- | --- | --- | --- | --- | --- |
|  | MTAP intact  N=4,519 | MTAP loss  N=1,228 | P value | MTAP intact  N=1,611 | MTAP loss  N=302 | P value | MTAP intact  N=562 | MTAP loss  N=12 | P value | MTAP intact  N=13.522 | MTAP loss  N=154 | | P value | MTAP intact  N=478 | MTAP loss  N=57 | P value |
| PD-L1  Low Positive (%) | 1,405  (31.1) | 418  (34.0) | 0.121 | 409  (25.4) | 53  (17.5) | 0.013 | 112  (19.9) | 3  (25.0) | 1.000 | 1,920  (14.2) | | 28  (18.2) | 0.295 | 86  (18.0) | 7  (12.3) | 0.523 |
| PD-L1  High Positive (%) | 262  (5.8) | 97  (7.9) | 0.024 | 93  (5.8) | 17  (5.6) | ns | 21  (3.7) | 1  (8.3) | 1.000 | 189  (1.4) | | 9  (5.8) | 0.002 | 26  (5.4) | 1  (1.8) | 0.523 |
| PD-L1  Any Positive (%) | 1,667  (36.9) | 515  (41.9) | 0.005 | 502  (31.2) | 70  (23.2) | 0.017 | 133  (23.7) | 4  (33.3) | 1.000 | 2,109  (15.6) | | 37  (24.0) | 0.022 | 112  (23.4) | 8  (14.1) | X |
| PD-L1  Negative (%) | 2,852  (63.1) | 713  (58.1) | 0.005 | 1,109  (68.8) | 232  (76.8) | 0.017 | 429  (76.3) | 8  (66.7) | 1.000 | 11,413  (84.4) | | 117  (76.0) | 0.022 | 366  (76.6) | 49  (86.0) | 0.267 |

*Legend: PDAC: pancreatic ductal adenocarcinoma; IHCC: intrahepatic cholangiocarcinoma; HCC: hepatocellular carcinoma; CRC: colorectal carcinoma; GEAC: gastroesophageal adenocarcinoma.*

**Supplementary Table 2: Distribution of COSMIC trinucleotide signatures according to MTAP tumor status in Genomics only cohort**

|  | **PDAC** | | | **IHCC** | | | **HCC** | | | **CRC** | | | | **GEAC** | | | |
| --- | --- | --- | --- | --- | --- | --- | --- | --- | --- | --- | --- | --- | --- | --- | --- | --- | --- |
|  | MTAP intact  N=213 | MTAP loss  N=48 | P value | MTAP intact  N=185 | MTAP loss  N=23 | P value | MTAP intact  N=87 | MTAP loss  N=1 | P value | MTAP intact  N= 3,856 | MTAP loss  N= 41 | | P value | MTAP intact  N= 733 | MTAP loss  N= 47 | P value |  |
| **MMR** | 100  (46.9) | 8  (16.7) | 0.0006 | 93  (50.3) | 2  (8.7) | 0.0006 | 12  (13.8) | 0  (0) | 1.000 | 2,329  (60.4) | | 14  (34.1) | 0.005 | 298  (40.7) | 13  (27.7) | 0.214 |  |
| **APOBEC** | 12  (5.6) | 8  (16.7) | 0.044 | 18  (9.7) | 5  (21.7) | 0.289 | 0  (0) | 0  (0) | 1.000 | 50  (1.3) | | 3  (7.3) | 0.054 | 28  (3.8) | 5  (10.6) | 0.132 |  |
| **UV** | 1  (0.5) | 0  (0) | 1.000 | 2  (1.1) | 0  (0) | 1.000 | 1  (1.2) | 0  (0) | 1.000 | 8  (0.21) | | 2  (4.9) | 0.015 | 6  (0.8) | 1  (2.1) | 0.523 |  |
| **Tobacco** | 15  (7.0) | 3  (6.3) | 1.000 | 14  (7.6) | 3  (13.0) | 0.623 | 5  (5.7) | 0  (0) | 1.000 | 77  (2.0) | | 2  (4.9) | 0.332 | 18  (2.5) | 1  (2.1) | 1.000 |  |
| **Alkylating** | 0  (0) | 1  (2.1) | 0.346 | 0  (0) | 0  (0) | 1.000 | 0  (0) | 0  (0) | 1.000 | 3  (0.078) | | 0  (0) | 1.000 | 5  (0.7) | 1  (2.1) | 0.523 |  |
| **POLE** | 2  (0.9) | 0  (0) | 1.000 | 1  (0.5) | 0  (0) | 1.000 | 0  (0) | 0  (0) | 1.000 | 147  (3.8) | | 0  (0) | 0.560 | 0  (0) | 0  (0) | 1.000 |  |

*Legend: PDAC: pancreatic ductal adenocarcinoma; IHCC: intrahepatic cholangiocarcinoma; HCC: hepatocellular carcinoma; CRC: colorectal carcinoma; GEAC: gastroesophageal adenocarcinoma; MMR: mismatch repair; APOBEC: Apolipoprotein B mRNA editing catalytic polypeptide-like; UV: ultraviolet radiation; POLE: Polymerase epsilon.*

**Supplementary Table 3: Genomic characteristics of patients in Clinical outcomes cohort**

| **Patient Characteristics** | **IHCC**  N= 70 | | **PDAC**  N=32 | |
| --- | --- | --- | --- | --- |
|  | ***MTAP-*intact**  N= 49 | ***MTAP-*loss**  N= 21 | ***MTAP-*intact**  N= 11 | ***MTAP-*loss**  N= 21 |
| **AJCC v8 stage at diagnosis (%)** |  |  |  |  |
| IV | 49 (100) | 21 (100) | 11 (100) | 21 (100) |
| **Immunotherapy biomarkers (%)** |  |  |  |  |
| Microsatellite instability status  High  Stable  Missing | 1 (2.0)  32 65.3)  16 (32.7) | 0 (0)  21 (100)  0 (0) | 0 (0)  7 (63.6)  4 (36.4) | 0 (0)  19 (90.4)  2 (9.5) |
| PD-L1 CPS  0  <1  >1  Missing | 2 (4.1)  1 (2.0)  2 (4.1)  44 (90.0) | 1 (4.8)  2 (9.5)  0 (0)  18 (85.7) | 3 (27.3)  3 (27.3)  0 (0)  5 (45.4) | 2 (9.5)  0 (0)  2 (9.5)  17 (81.0) |
| TMB  Low, <10 Mut/Mb  High, ≥10 Mut/Mb  Missing | 17 (34.7)  2 (4.1)  30 (61.2) | 20 (95.2)  0 (0)  1 (4.8) | 11 (100)  0 (0)  0 (0) | 16 (76.2)  1 (4.8)  4 (19.0) |
| **Co-altered genes** |  |  |  |  |
| *AKT* | 0 (0) | 0 (0) | 1 (9.1) | 0 (0) |
| *ARID1A* | 8 (16.3) | 1 (4.8) | 1 (9.1) | 1 (4.8) |
| *ATM* | 3 (6.1) | 2 (9.5) | 0 (0) | 0 (0) |
| *AXL* | 0 (0) | 0 (0) | 1 (9.1) | 0 (0) |
| *BAP1* | 4 (8.2) | 4 (19.0) | 0 (0) | 0 (0) |
| *BRAF* | 4 (8.2) | 0 (0) | 0 (0) | 0 (0) |
| *BRCA2* | 0 (0) | 0 (0) | 1 (9.1) | 3 (14.3) |
| *CCNE1* | 0 (0) | 2 (9.5) | 0 (0) | 0 (0) |
| *CDK6* | 0 (0) | 0 (0) | 2 (18.2) | 0 (0) |
| *CDKN2A* | 8 (16.3) | 19 (90.5) | 7 (63.6) | 18 (85.7) |
| *CDKN2B* | 4 (8.2) | 16 (76.2) | 5 (45.5) | 18 (85.7) |
| *CHEK2* | 0 (0) | 0 (0) | 1 (9.1) | 0 (0) |
| *ERBB2* | 4 (8.2) | 3 (14.3) | 0 (0) | 0 (0) |
| *ERBB3* | 0 (0) | 0 (0) | 0 (0) | 2 (9.5) |
| *FGF19* | 2 (4.1) | 2 (9.5) | 0 (0) | 0 (0) |
| *FGFR2* | 15 (30.6) | 4 (19.0) | 0 (0) | 0 (0) |
| *GATA6* | 0 (0) | 0 (0) | 1 (9.1) | 2 (9.5) |
| *GNAS* | 0 (0) | 0 (0) | 2 (18.2) | 0 (0) |
| *IDH1* | 9 (18.4) | 0 (0) | 0 (0) | 0 (0) |
| *KDM6A* | 0 (0) | 0 (0) | 1 (9.1) | 0 (0) |
| *KRAS* | 7 (14.3) | 3 (14.3) | 11 (100) | 17 (81.0) |
| *MDM2* | 4 (8.2) | 1 (4.8) | 0 (0) | 0 (0) |
| *MLL2* | 0 (0) | 0 (0) | 0 (0) | 3 (14.3) |
| *MYC* | 7 (14.3) | 1 (4.8) | 1 (9.1) | 0 (0) |
| *NRAS* | 2 (4.1) | 3 (14.3) | 0 (0) | 0 (0) |
| *PBMR1* | 3 (6.1) | 0 (0) | 0 (0) | 0 (0) |
| *PIK3CA* | 5 (10.2) | 2 (9.5) | 0 (0) | 1 (4.8) |
| *RB1* | 0 (0) | 0 (0) | 1 (9.1) | 2 (9.5) |
| *SMAD4* | 1 (2.0) | 1 (4.8) | 0 (0) | 4 (19.0) |
| *STK11* | 0 (0) | 2 (9.5) | 0 (0) | 0 (0) |
| *TET2* | 0 (0) | 0 (0) | 1 (9.1) | 0 (0) |
| *TP53* | 12 (24.5) | 3 (14.3) | 10 (90.9) | 13 (61.9) |
| *U2AF1* | 0 (0) | 0 (0) | 0 (0) | 2 (9.5) |

**Supplementary Table 4: Multivariate analysis of overall survival in advanced pancreatic cancer and cholangiocarcinoma patients from the Clinical outcomes cohort**

| **Pancreatic Cancer** | | | | | | | |
| --- | --- | --- | --- | --- | --- | --- | --- |
| **Parameter** | | **Parameter Estimate** | **Standard Error** | **Hazard Ratio** | **95% Hazard Ratio Confidence Limits** | | **P-Value** |
| *ARID1A* | Altered versus intact | 2.61 | 1.01 | 13.59 | 1.89 | 97.72 | 0.010 |
| *U2AF1* | Altered versus intact | 2.70 | 1.28 | 14.80 | 1.24 | 177.32 | 0.033 |
| **Intrahepatic Cholangiocarcinoma** | | | | | | | |
| **Parameter** | | **Parameter Estimate** | **Standard Error** | **Hazard Ratio** | **95% Hazard Ratio Confidence Limits** | | **P-Value** |
| *CDKN2A* | Altered versus intact | 0.77 | 0.36 | 2.15 | 1.06 | 4.40 | 0.035 |
| *CCNE1* | Altered versus intact | 2.18 | 1.04 | 8.86 | 1.16 | 67.69 | 0.035 |
| *MYC* | Altered versus intact | 1.10 | 0.44 | 3.01 | 1.28 | 7.10 | 0.012 |
